# Intron Evolution in Saccharomycetaceae

**DOI:** 10.1093/gbe/evu196

**Published:** 2014-09-09

**Authors:** Katarzyna B. Hooks, Daniela Delneri, Sam Griffiths-Jones

**Affiliations:** ^1^Faculty of Life Sciences, University of Manchester, United Kingdom; ^2^U1053 INSERM, Université de Bordeaux, France

**Keywords:** intron loss, yeast, fungi

## Abstract

Introns in protein-coding genes are very rare in hemiascomycetous yeast genomes. It has been suggested that these species have experienced extensive intron loss during their evolution from the postulated intron-rich fungal ancestor. However, no intron-devoid yeast species have been identified and some of the introns remaining within the genomes of intron-poor species, such as *Saccharomyces cerevisiae*, appear to be beneficial during growth under stress conditions. In order to reveal the pattern of intron retention within intron-poor yeast species and better understand the mechanisms of intron evolution, we generated a comprehensive set of 250 orthologous introns in the 20 species that comprise the Saccharomycetaceae, by analyzing RNA deep-sequencing data and alignments of intron-containing genes. Analysis of these intron sets shows that intron loss is at least two orders of magnitude more frequent than intron gain. Fine mapping of intron positions shows that intron sliding is rare, and that introns are almost always removed without changing the primary sequence of the encoded protein. The latter finding is consistent with the prevailing view that homologous recombination between reverse-transcribed mature mRNAs and the corresponding genomic locus is the primary mechanism of intron loss. However, we also find evidence that loss of a small number of introns is mediated by micro-homology, and that the number of intron losses is diminished in yeast species that have lost the microhomology end joining and nonhomologous end joining machinery.

## Introduction

The origin and evolution of introns in eukaryotic genomes are intensively debated topics (Rogozin et al. 2012). Two long-standing theories have been proposed to describe the origin of introns: The “introns early” and “introns late” hypotheses. These theories placed the origin of introns before and after the Eukaryota–Prokaryota split, respectively (Doolittle 1978; Stoltzfus et al. 1994). More recently, the “introns first” hypothesis, building on the “RNA world” concept, has attributed the origin of introns to self-splicing RNA molecules that were evolutionary forerunners of protein-coding RNAs (Poole et al. 1998). Almost all known eukaryotes possess at least a few introns and the machinery required to splice them. However, it is generally considered unlikely that the Eubacteria and Archea ancestors ever possessed a spliceosome (Stoltzfus et al. 1994). The current consensus therefore is a version of the introns late hypothesis: that introns evolved early within the eukaryotic lineage (Rogozin et al. 2012). The most likely scenario is that the emergence of spliceosomal introns from founder group II self-splicing introns happened shortly after the endosymbiosis of the protomitochondrial bacteria by the archeal host (Martin and Koonin 2006). However, neither the introns early nor introns first theories have been conclusively discounted (Penny et al. 2009).

Analyses of intron distribution among different eukaryotes have pinpointed a surprisingly high percentage of shared intron positions in orthologous genes (Fedorov et al. 2002; Rogozin et al. 2003). Because some introns are present in the same positions in genes in plants, animals, and fungi, these studies concluded that they must have been present in the last eukaryotic common ancestor. Additionally, intron loss appears to be more common than gain throughout eukaryotic evolution. In particular, a study restricted to fungal introns (Stajich et al. 2007) hypothesized an intron-rich fungal ancestor, from which extensive intron loss has occurred in the hemiascomycetous yeasts. Indeed, there is very little evidence of intron gain in any lineage, but this is likely, at least in part, to be due to the difficulty in detecting such events. For example, in mammalian genomes, no unambiguous intron gains have been identified (Roy et al. 2003). A study of nematode introns found evidence for 122 gains (Coghlan and Wolfe 2004), but later sequencing of multiple worm species identified orthologous introns that push the intron origins further back in time (Roy and Penny 2006). More recently, strong evidence for intron gain has been obtained using closely related fungal species (Torriani et al. 2011) and sequenced isolates of *Daphnia pulex* (Li et al. 2009). Both of these studies identify a number of unique, transient introns that have not yet been fixed within their respective populations.

**Fig. 1.—:**
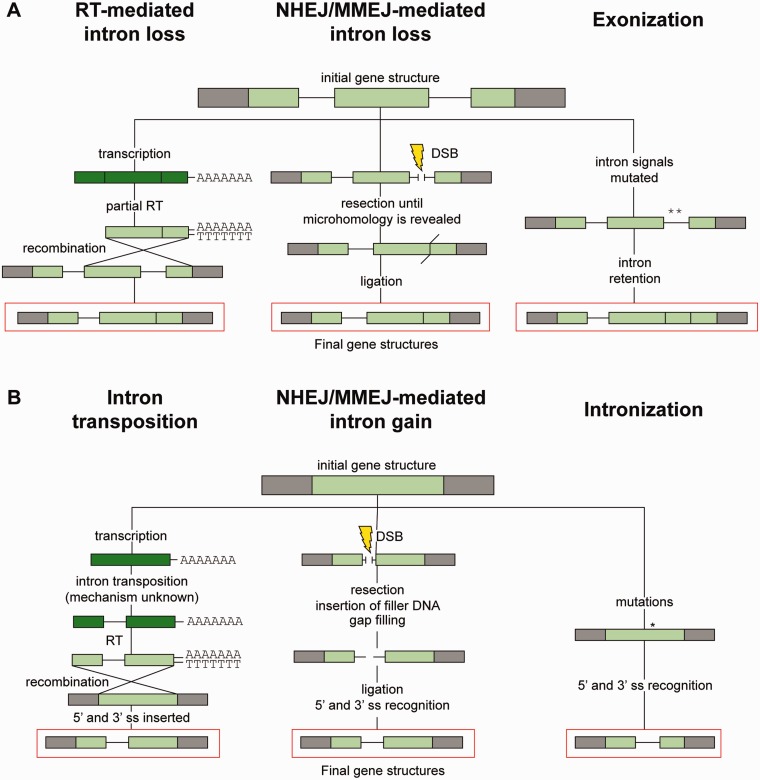
Postulated mechanisms of intron loss and gain. Simplified mechanisms of loss (*A*) and gain (*B*) are presented. Genomic contexts are represented as gray boxes, exons in DNA as light green boxes, exons in RNA as dark green, and introns as thin lines joining exons. The resulting gene structure for each mechanism is framed in red. Note that the NHEJ/MMEJ-mediated mechanism does not necessarily lead to perfect intron gain or loss (not shown). RT, reverse transcription.

Various mechanisms of intron loss and gain have been proposed (fig. 1). The most plausible intron loss mechanism is homologous recombination between reverse-transcribed mature mRNA and the genomic locus (Mourier and Jeffares 2003). This model is attractive in that it can explain the visible bias of intron position toward the 5′-end of genes of intron-poor species. It has also been proposed that introns might be lost by “genomic deletion,” which involves enzymes from nonhomologous- and microhomology-mediated end-joining DNA repair (NHEJ and MMEJ). It was initially assumed that intron loss promoted by double-strand breaks (DSB) would lead to imprecise intron deletions (Roy and Gilbert 2005) but it was since noted that the presence of AG|GT consensus in both 5′- and 3′-splice sites could serve as a microhomology and thus facilitate exact intron deletion (Hu 2006; Farlow et al. 2011; van Schendel and Tijsterman 2013). Although the number of observed intron gain events is low, various mechanisms have been suggested to explain them, including insertion of a group II intron (Martin and Koonin 2006), exon intronization (Gao and Lynch 2009), intron retrotransposition (Torriani et al. 2011), and DSB repair (Li et al. 2009).

Hemiascomycetous yeast belong to one of the best-studied eukaryotic clades, frequently used for comparative and evolutionary studies (Cliften et al. 2001; Dujon et al. 2004; Gordon et al. 2009). The clade can be divided into three groups: Early branching yeast species such as *Yarrowia lipolytica*, the “CTG group,” which translates CTG as serine instead of leucine, and Saccharomycetaceae, which includes *Saccharomyces cerevisiae*. The ancestor of *S. cerevisiae* underwent a whole-genome duplication (WGD), followed by extensive loss of one copy of most paralogous genes (Wolfe and Shields 1997). All hemiascomycetous yeast have experienced extensive intron loss, and on average introns are found in only 5 % of their genes (Neuveglise et al. 2011). Introns in *S. cerevisiae* have been well annotated due to the extensive efforts of the yeast scientific community (Saccharomyces Genome Database [SGD]). However, introns in most other yeast species are poorly annotated, and intron analyses therefore rely either on automatic annotation or small manually curated sets of intron orthologs. This impedes the estimation of intron number and subsequent evolutionary studies. Nonetheless, even with limited intron information, interesting evolutionary observations have been made for the Saccharomycotina subphylum*.* For example, hemiascomycetous yeasts contain only the U2 spliceosome; all the components specific to U12 spliceosome have been lost in the Ascomycota (Russell et al. 2006; Bartschat and Samuelsson 2010). Unlike in mammals, the intronic elements involved in splicing obey a strict consensus sequence pattern in Saccharomycetaceae: GTATGT for the 5′-splice site (5′-ss), TACTAAC for the branch point (BP) (Bon et al. 2003), and the 3′-splice site (3′-ss) always finishes in AG. However, yeasts lack the usual poly-T track between the BP and 3′-ss (Irimia and Roy 2008); instead the 3′-ss is defined by the distance from BP, which can vary between species (Neuveglise et al. 2011). Neuveglise et al. (2011) suggested that the BP-3′-ss distance is constrained by the *U2AF1* splicing factor, and that species that have lost *U2AF1*, such as *S. cerevisiae*, *Candida glabrata*, and *Kluyveromyces lactis*, exhibit a longer BP-3′-ss distance.

In this study, we present a comprehensive evaluation of intron evolution in the Saccharomycetaceae by exploiting the high-quality orthologous relationships provided by the Yeast Gene Order Browser (YGOB) (Byrne and Wolfe 2005), together with RNA deep-sequencing data. We constructed multiple sequence alignments of 235 intron-containing genes in 20 Saccharomycetaceae species in order to determine the exact fate of each intron-containing gene after the WGD. We found that intron loss events are at least two orders of magnitude more common than gains. Intron loss appears to be branch- and species-specific, and is usually “perfect,” suggesting that loss has resulted from replacement of the original intron-containing gene with the intron-less cDNA. Furthermore, we found clear examples of intron loss accompanied by insertion of additional codons, at least one case of intron sliding, and we have shown that the uncoupling of snoRNAs from introns of some genes was enabled by WGD. Lastly, an analysis of intron conservation in ribosomal protein genes among 12 tested species allowed us to draw conclusions about how ribosomal protein gene (RPG) intron function might have evolved. We discuss the prominent mechanisms of evolution of yeast introns in relation to our findings.

## Materials and Methods

### RNAseq

*Saccharomyces cerevisiae* strain BY4741, *Saccharomyces kudriavzevii*, and *Naumovia castellii* were cultured in standard YPD media in 30 °C and *Saccharomyces uvarum* NCYC 2669 at 28 °C with shaking at 200 rpm, to an absorbance of 0.5 at 600 nm. RNA of *S. cerevisiae* was extracted using Trizol (Invitrogen, UK), precipitated in lithium chloride (Ambion, UK), washed twice with 70% ethanol and the pellet resuspended in dH_2_O. RNA of the other three species was extracted using Qiagen RNA extraction kit according to manufacturer’s instructions. Ten micrograms of total RNA from each *S. cerevisiae*, *S. kudriavzevii*, *S. uvarum*, and *N. castellii* was processed with the RiboMinus Transcriptome Isolation Kit for Yeast and Bacteria (Invitrogen) to deplete the rRNA. cDNA libraries for each species were constructed and sequenced using the SOLiD 4.0 System from Life Technologies according to the standard manufacturer’s protocol. *Saccharomyces cerevisiae* cDNA was deposited on one-quadrant of a slide with one other barcoded library (not presented here). The other three libraries were barcoded and deposited on one-quadrant of a slide. Sequencing yielded a total of 77,286,181 reads for *S. cerevisiae*, 23,993,647 reads for *S. uvarum*, 6,287,432 reads for *S. kudriavzevii*, and 7,909,274 reads for *N. castellii*, all of 50 bps. Raw reads were filtered to obtain only reads with an average quality >20 using the approach described by Sasson and Michael (2010). Reads were mapped to genomes downloaded from YGOB version 7 with Bowtie 0.12.7 (allowing up to two mismatches, and retaining only reads mapping to one location in the genome: -v 2 -m 1). Splice junctions were identified using Tophat with default parameters and defined intron size between 49 and 1,050 bp (-i 49 -I 1050; representing the minimum and maximum lengths of introns in *S. cerevisiae* genes according to SGD, release 64) (Trapnell et al. 2009). The RNAseq data are deposited in the Gene Expression Omnibus database (accession number GSE58884).

### Intron Alignments

Annotations for 306 introns in 286 genes for *S. cerevisiae* were extracted from SGD (http://www.yeastgenome.org/, release 64, last accessed September 1, 2014). The orthologs of the intron-containing genes in the *S. cerevisiae* clade were retrieved from the YGOB Pillar file (http://ygob.ucd.ie/, version 7, last accessed September 1, 2014) (Byrne and Wolfe 2005), which presents the gene homology among species. In order to obtain alignments of intron-containing genes, we first used sequences for *S. cerevisiae*, *Saccharomyces mikatae*, *S. kudriavzevii*, and *S. uvarum* to create a seed alignment with mLAGAN (Brudno et al. 2003), and then manually edited it using RALEE (Griffiths-Jones 2005) to ensure the correct alignment of both splice sites and BPs. DNA seed alignments were used to search for intron-containing genes in all other YGOB species using HMMER 3.1b1 (Wheeler and Eddy 2013). Briefly, hmmbuild was used to build the profile HMM from sequence alignments, and then nhmmer was used to search the profile against a DNA database containing all sequences from YGOB. Lists of hits were manually inspected, and potential matches were extracted and aligned against the seed alignment using hmmalign. An iterative procedure of HMMER search, alignment, manual inspection, and editing allowed us to construct full gene alignments, with annotated intron boundaries and BPs. These alignments allowed us to confirm intron presence, perfect deletion, or imperfect deletion resulting in the removal or insertion of additional codons. Where the alignment did not allow us to conclusively prove intron presence, we assumed the intron was removed by an unknown mechanism and, thus, counted it as an intron loss for the subsequent phylogenetic analysis. In the cases where a significant portion of a gene sequence was missing from the assembly, the specific gene sequence was excluded from the alignment and subsequent analysis.

To detect orthologs of intronic snoRNAs, we extracted the portion of each intron alignment that corresponded to a snoRNA annotation in *S. cerevisiae* (SGD). We then used the resulting alignments as seeds for iterative HMMER searches as described above. We analysed the sequence surrounding the potential snoRNA matches to establish if they are encoded within introns of paralogous genes. Lastly, we used syntenic information provided by YGOB to establish if the snoRNA hits lie in regions paralogous to those included in the seed alignment.

### Phylogenetic Analysis

Syntenic relationships between intron-containing genes were extracted from YGOB. Paralogous genes arising from WGD were grouped to represent one ancestral gene, and multiple introns in the same gene were treated separately. The phylogenetic tree used to map intron gains and losses was taken from YGOB (simplified version from Hedtke et al. 2006). An additional branch reflecting the WGD loci was added creating a tree with both A and B loci for all post-WGD species. For the analysis of each intron alignment, the tree was pruned to include only the species found in that alignment. For each ancestral intron, gains and losses were placed automatically on the pruned yeast tree using the Dollo parsimony method (Farris 1977) implemented in the Dollop script from the Phylip package. All assignments were manually inspected. We assumed that the common ancestor had almost all introns currently present in tested species (Stajich et al. 2007). We therefore manually inspected all assigned intron gains, and 3 out of 11 were converted into losses on alternative branches of the tree. After the event assignment was complete, we summed up all the instances of intron loss and gain on a simplified tree without the duplicated branch, so that each branch in post-WGD species represents a sum for corresponding branches in A and B loci.

## Results

### *Conserved Splicing in* Saccharomyces Sensu Stricto *Species*

We used RNA deep sequencing experiments to annotate intron positions in *S. cerevisiae*, *S. kudriavzevii*, *S*. *uvarum*, and *N. castellii*. We predicted splice junctions from RNAseq data using Tophat, and confirmed the presence of the canonical splice signals (GTATGT/AG) and BP (TACTAAC) sequences that are indicative of Saccharomycetaceae introns. We found 216, 163, 200, and 155 predicted introns in *S. cerevisiae*, *S. kudriavzevii*, *S. uvarum*, and *N. castellii*, respectively. Of these, three introns in *S. cerevisiae*, four in *S. uvarum* and ten in *N. castellii* are novel—not present in the latest annotation in SGD—although some have been previously experimentally validated (table 1). Novel introns in *N. castellii* are not present in *Saccharomyces* sensu stricto genomes: Eight appear to have been lost through cDNA replacement and two are located in the out-paralogs of *S. cerevisiae RPL29* and *RPL39* genes.

**Table 1:** Novel Introns Predicted from RNAseq by Tophat

Gene	Predicted Introns (This Study)				Previous Studies
	*S.cer*	*S.kud*	*S.uva*	*N.cas*	*S.cer*
*FES1*	G,R	G	G	G	Yassour et al. (2009)
*RPS22B*/snR44	G,R	G	G,R	–	Yassour et al. (2009)
*PUS2* (5′-UTR)	G,R	G	G,R	G	None
*YTA12* (5′-UTR)	–	G	G,R	–	None
YMR147W/YMR148W	G	G	G,R	–	Miura et al. (2006)
*GTR2*	–	–	–	G,R	None
YKL033W-A	–	–	–	G,R	None
*RRN3*	–	–	–	G,R	None
*SEC22*	–	–	–	G,R	None
*VAN1*	–	–	–	G,R	None
*QCR6*	–	–	–	G,R	None
*PMP3*	–	–	–	G,R	None
*ARC18*	–	–	–	G,R	None
*RPL29* outparalog	–	–	–	G,R	None
*RPL39* outparalog	–	–	–	G,R	None

Note.–Species names: *S.cer*, *S. cerevisiae*; *S.kud*, *S. kudriavzevii*; *S.uva*, *S. uvarum*; *N.cas*, *N castellii*; –, no intron in the genome; G, intron found in the genome; R, intron predicted by RNAseq.

**Fig. 2.—:**
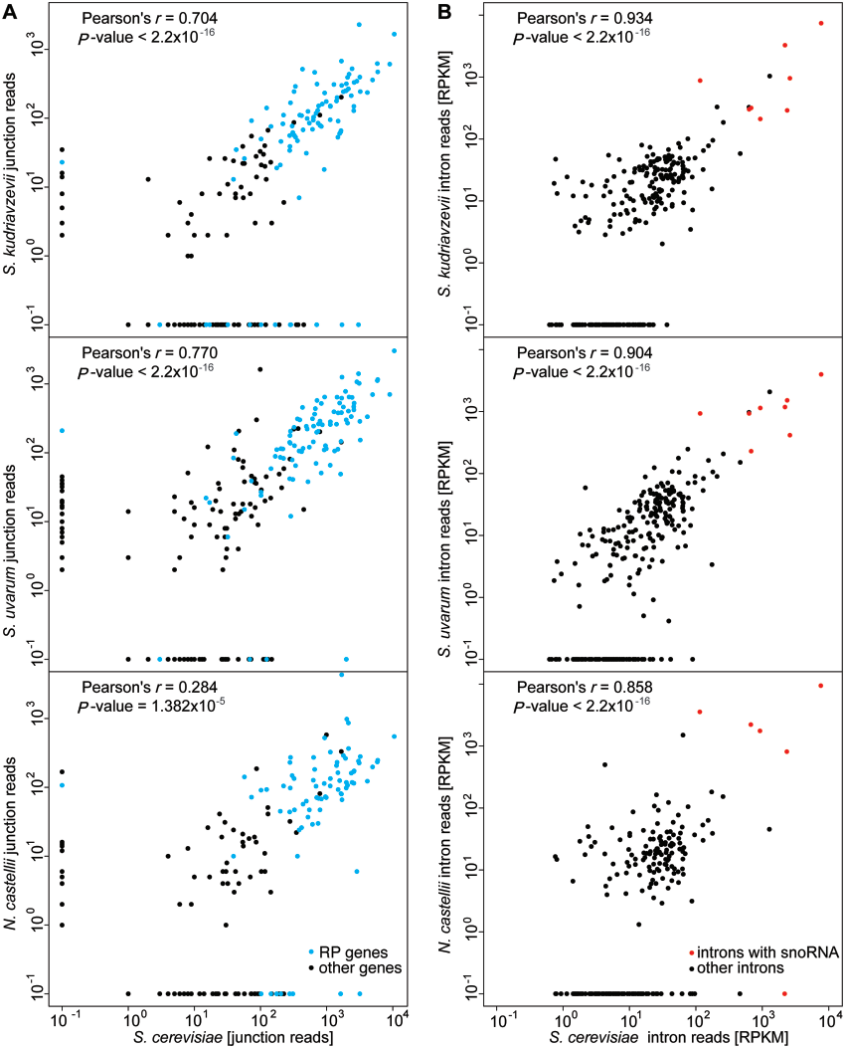
Conservation of intron splicing and expression in RNAseq data. Number of reads spanning confirmed junctions (*A*) and intron expression estimated by the number of reads per kb per million mapped (*B*) *Saccharomyces kudriavzevii*, *S. uvarum,* and *N. castellii* RNAseq is compared with corresponding data from *S. cerevisiae*. RP genes are indicated in blue and snoRNA-containing introns in red. Pearson’s product-moment correlation and *P* value are shown above each graph.

Next, we counted the reads spanning each identified junction in *S. cerevisiae* and compared the number with the orthologous junctions of three other species. We found strong correlations between normalized junction read counts within the *Saccharomyces* sensu stricto group, indicating similar number of spliced transcripts (fig. 2*A*). Comparing the numbers of reads mapped to each intron among the various species revealed an even stronger correlation between species (fig. 2*B*). These results provide preliminary evidence that intron splicing and expression is generally highly conserved among the *Saccharomyces* sensu stricto species, but more specific investigation of levels of intron splicing is warranted.

### Identifying Orthologous Introns

We built multiple sequence alignments of intron-containing genes using the annotated gene set from *S. cerevisiae* (SGD) and introns predicted from the RNAseq data obtained for *S. cerevisiae*, *S*. *uvarum*, and *N. castellii*. We extracted the orthologous genes from YGOB and investigated the presence of introns for those species in which the host genes were present. Manual refinement of these alignments highlighted five additional unannotated introns in genes *HOP2*, *CGI121*, YPR170W-B, YPR153W, and YPL109C, of which only the *HOP2* intron is present in *S. cerevisiae* (the *HOP2* intron is not yet in SGD, but was recently described by Chan et al. [2014]). Thus the total number of ancestral intron sites used for the subsequent phylogenetic analysis was 250. The number of introns identified in each species is shown in table 2. We found 15 more introns in *C. glabrata* than estimated previously (Dujon et al. 2004; Gabaldon et al. 2013), a similar number in *Zygosaccharomyces rouxii* and in *K. lactis*, and around 75% of those previously predicted in *Eremothecium gossypii*, *Lachancea kluyveri*, and *Lachancea thermotolerans* (Neuveglise et al. 2011). Because we focused on introns with orthologs in *S. cerevisiae*, it is expected that we identify only a subset of the introns annotated in the more distant species.

**Table 2:** Number of Introns Found in Each Species

Species	Number of Introns Found	Number of Previously Reported
*Vanderwaltozyma polyspora*	193	–
*Tetrapisispora phaffii*	165	–
*Tetrapisispora blattae*	172	–
*Naumovozyma dairenensis*	234	–
*Naumovia castellii*	265	–
*Kazachstania naganishii*	185	–
*Kazachstania africana*	226	–
*Glabrata glabrata*	144	105^a^, 129^b^^,^^c^
*Saccharomyces uvarum*	287	–
*Saccharomyces kudriavzevii*	288	–
*Saccharomyces mikatae*	288	–
*Saccharomyces cerevisiae*	290	280^a^, 296^c^, 306^d^
*Zygosaccharomyces Rouxii*	173	162^a^, 168^c^
*Torulaspora delbrueckii*	211	–
*Kluyveromyces lactis*	174	129^a^, 176^c^
*Eremothecium gossypii*	197	259^a^, 222^c^
*Eremothecium cymbalariae*	204	–
*Lachancea kluyveri*	241	335^a^, 321^c^
*Lachancea thermotolerans*	226	296^a^, 285^c^
*Lachancea waltii*	222	–
Total	4,385	–

^a^Genosplicing (http://genome.jouy.inra.fr/genosplicing/patterns.html, last accessed September 1, 2014).

^b^Gabaldon et al. (2013).

^c^Neuveglise et al. (2011).

^d^Saccharomyces genome database (http://www.yeastgenome.org, last accessed September 1, 2014).

**Fig. 3.—:**
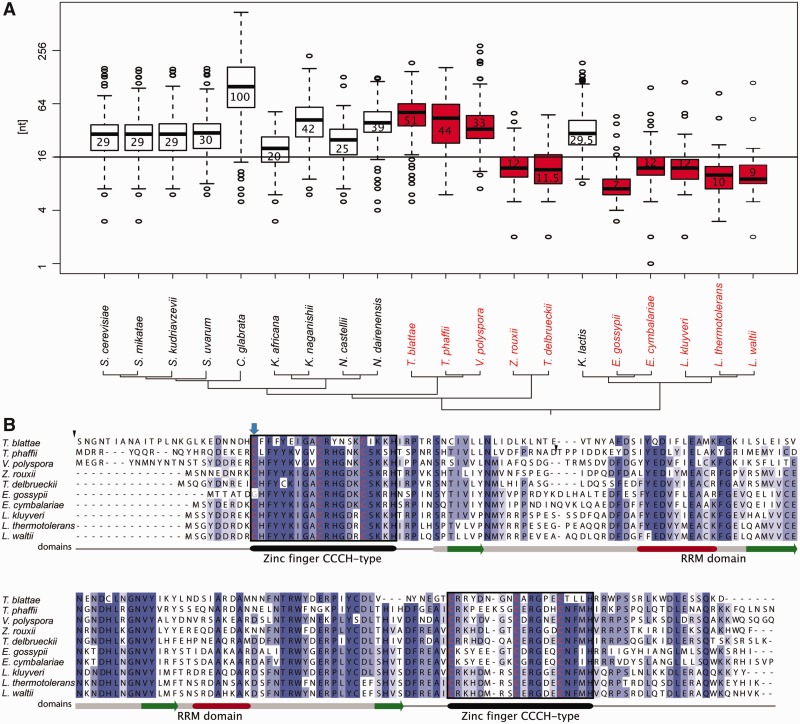
S2 distance and U2AF1 gene conservation. (*A*) Boxplot of log_2_ S2 distance based on constructed alignments. Species indicated in red contain the copy of U2AF1 gene. The mean S2 value for each species is displayed. (*B*) Alignment of the U2AF1 protein with indicated insertions (black triangles), the intron position (blue arrow) and protein domain structure; green arrows, β-strands; red boxes, α-helices.

Based on the multiple sequence alignments of intron sequences (see Supplementary Material online) we investigated the differences in the distance between the BP and the 3′-splice site (referred to as the S2 distance) among 20 species. As reported previously, we found increased S2 distances in *C. glabrata*, *K. lactis* and to lesser extent in *Saccharomyces* sensu stricto, *Kazachstania* sp. and *Naumovozyma* sp. All those species have lost the *U2AF1* splicing factor postulated to be correlated with short S2 distances (Neuveglise et al. 2011). However, *Vanderwaltozyma polyspora*, *Tetrapisispora phaffii*, and *Tetrapisispora blattae* also have a median S2 distance higher than 30 despite possessing the *U2AF1* gene (fig. 3*A*). We examined the *U2AF1* gene and protein structure in detail, and found that *T. blattae* and *T. phaffii* are the only species without the intron splitting the initial cysteine codon of the first zinc finger domain. The *T. blattae* and *T. phaffii U2AF1* proteins also contain 105 and 60 amino acid insertions, respectively, but maintain all crucial domains (fig. 3*B*). The *V. polyspora U2AF1* gene contains the intron in the typical position and does not have extensive insertions. It was previously suggested that the loss of the *U2AF1* protein might be responsible for increased S2 distance (Neuveglise et al. 2011). Our data show that this is not the case, at least in the *Vanderwaltozyma–Tetrapisispora* group. A causal relationship between *U2AF1* loss and increased S2 distance is therefore unproven, but our data are consistent with an increased S2 distance allowing *U2AF1* to be lost in some species, rather than vice versa.

### Intron Gain and Loss

**Fig. 4.—:**
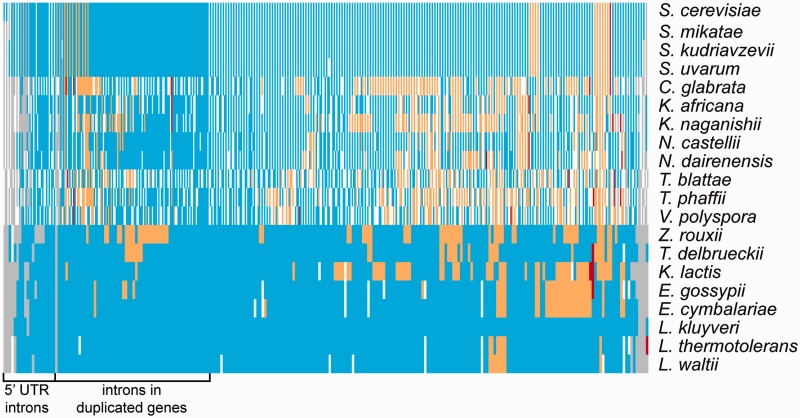
Heatmap showing intron evolution within YGOB species. Each row represents one of the species listed on the right. Each column corresponds to an ancestral intron. For post-WGD species columns are divided in two to represent the presence of the duplicate copies. Blue indicates intron presence, orange indicates loss by replacement of the gene with cDNA, red shows intron loss accompanied by additional codons inserted or deleted, and gray shows unknown state of intron.

**Fig. 5.—:**
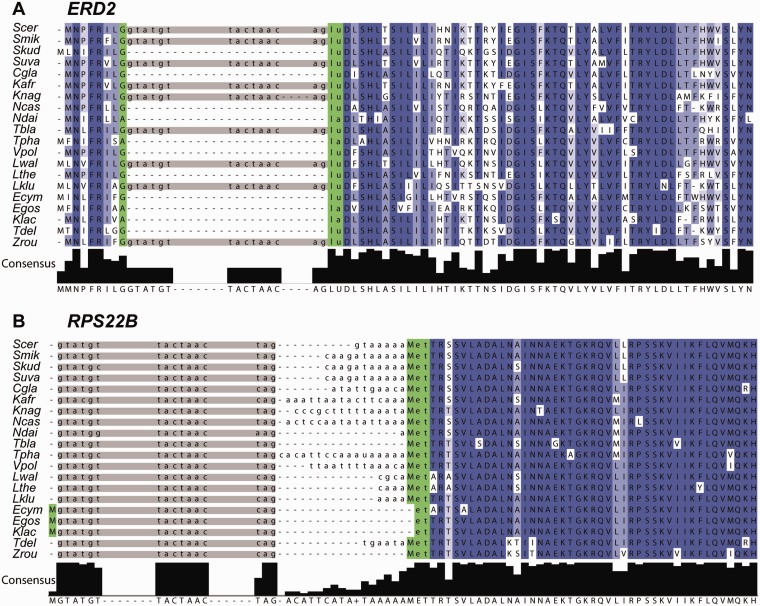
Intron alignments showing different outcomes of intron loss. Blue shading indicates protein conservation. Introns are represented by gray boxes with 5′-ss, BP and 3′-ss sequences shown. Species: *Saccharomyces cerevisiae*, *Saccharomyces mikatae*, *Saccharomyces kudriavzevii*, *Saccharomyces uvarum*, *Candida glabrata*, *Kazachstania africana*, *Kazachstania naganishii*, *Naumovozyma dairenensis*, *Naumovozyma castellii*, *Tetrapisispora blattae*, *Tetrapisispora phaffii*, *Vanderwaltozyma polyspora*, *Lachancea waltii*, *Lachancea thermotolerans*, *Lachancea kluyveri*, *Eremothecium cymbalariae*, *Eremothecium gossypii*, *Kluyveromyces lactis*, *Torulaspora delbrueckii*, and *Zygosaccharomyces rouxii*. (*A*) Protein alignment around the intron positions in the *ERD2* gene shows a typical example of multiple perfect intron losses. A codon interrupted by the intron is highlighted in green. (*B*) Protein alignment of the first exon of *RPS22B* showing 5′-UTR intron sliding into ORF in *E. cymbalariae*, *E. gossypii*, and *K. lactis*. The first methionine is indicated in green.

Across the 20 species and 235 ancestral genes, there were total of 5,553 “intron sites,” defined as sites that could contain an intron based on the presence of an intron at that position in at least one other species. In total, 1,168 of these intron sites are without an intron (0.21 introns missing per site per species) and 4,385 contained an intron (fig. 4, supplementary table S1, Supplementary Material online). The majority of missing introns (825/1,168, 71%) left the gene replaced with its cDNA version (adding or deleting a maximum of two codons, fig. 5*A*). There were 33 instances in 14 genes when intron removal was accompanied by a deletion or insertion of 3–40 codons. We found one clear case of intron sliding in the *RPS22B* gene in *Eremothecium cymbalariae*, *E. gossypii*, and *K. lactis*, where the 5′-UTR intron has been moved into the open reading frame (ORF) and is present after the initial A (fig. 5*B*). Finally, 310 additional introns, mainly in 5′-UTRs, appear to have been lost, but the mechanism of loss is unclear.

In order to understand better the evolutionary history of each intron, we placed intron gain and loss events on a phylogenetic tree using Dollo parsimony, and refined those placements by manual inspection. We found 630 evolutionary events of intron loss in 205 introns, with 159 introns lost more than once. Evidence for intron gains was much sparser: We identified only eight potential gain events in eight distinct genes. Introns in the 5′-UTR of both *MCR1* and *MTR2* were only present in *S. cerevisiae*. The *MRK1* intron was only found in *Saccharomyces* sensu stricto. An additional intron in the YPL109C gene has been gained in *L. thermotolerans* and *L. kluyveri*. We were able to identify divergent orthologous introns in *GCR1*, *USV1*, *YJR112W-A*, and an additional intron in *RPS22B*, in closely related species only, and thus it is unclear if these should be considered true gains. We conclude that intron gain events in yeast species occur at least two orders of magnitude less frequently than intron loss events. For 38 ancestral introns, all extant host genes in all species tested also contain an orthologous intron. Nineteen of those introns were found in RPGs.

**Fig. 6.—:**
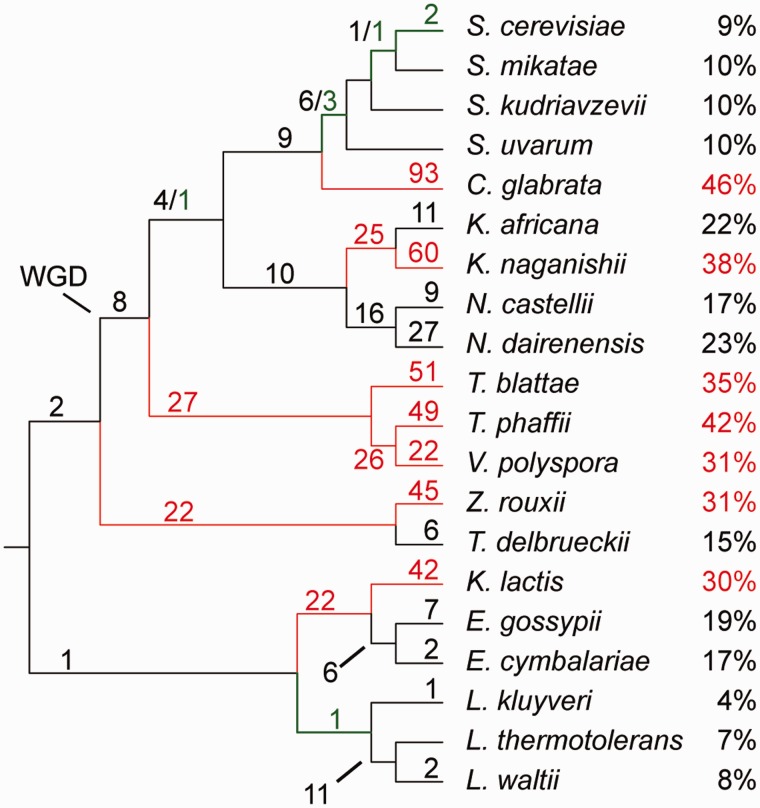
Tree displaying mapped intron gains and losses in YGOB species. The number above a branch indicates gain (green) and loss (red/black) on this branch. The number above a tip indicates gain/losses that happened in each species. Branches with more than 20 losses and species with more than 40 losses are displayed in red. The percentages on the right represent the proportion of genes with lost introns. The tree topology was taken from YGOB ver 7 (Byrne and Wolfe 2005) after Hedtke et al. (2006).

Some branches of the species tree have experienced more extensive intron loss than others (fig. 6). *Candida glabrata* has the highest apparent number of intron losses in this analysis: Of 266 genes orthologous to intron-containing genes in other species, *C. glabrata* contains only 144 introns. Additionally, *C. glabrata* appears to retain only one copy of each intron-containing gene after WGD (fig. 4), in contrast with other post-WGD species. Previous phylogenetic analyses placed *V. polyspora*, *Tetrapisispora phaffii*, and *T. blattae* most distant of the post-WGD species from *S. cerevisiae* (Hedtke et al. 2006; Scannell et al. 2007). Our results show *V. polyspora*, *Tetrapisispora phaffii*, and *T. blattae* have also experienced higher than average numbers of intron loss events. However, intron losses are not exclusive to post-WGD species, as exemplified by Z*. rouxii*, in which we identify 38 fewer introns than the sister species *Torulaspora delbrueckii.* At the other end of the spectrum, *L. kluyveri* has nine introns missing, with seven of them likely to be gains in other species and only one loss by an unknown mechanism on the *L. kluyveri* branch (figs. 4 and 6).

### Evolutionary History of Intronic snoRNAs

**Fig. 7.—:**
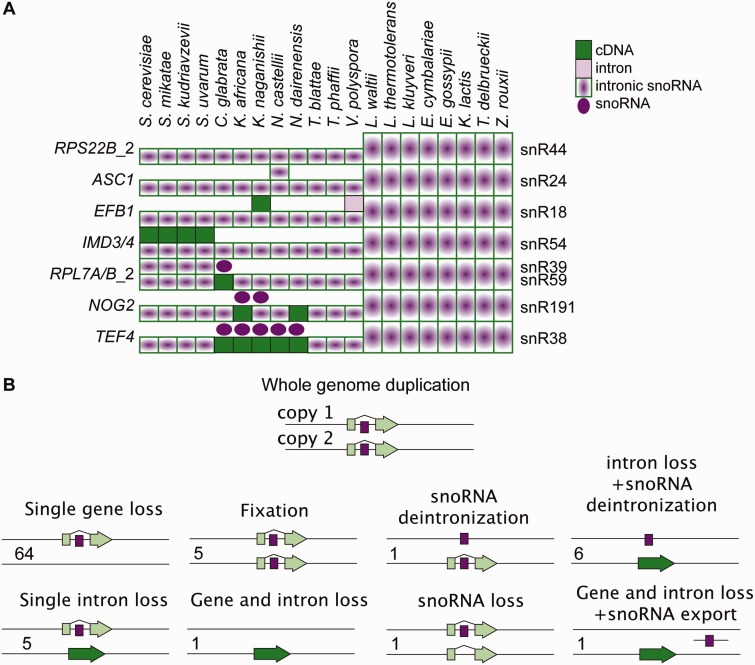
Evolution of intronic snoRNAs. (*A*) Diagram illustrating presence of intronic snoRNAs and their host genes in the *Saccharomyces* clade. Species are listed on the top, gene names are on the left, and intronic snoRNAs names are on the right. For post-WGD species, the state of both loci is shown according to the key in the top right corner. (*B*) Possible postduplication fates of genes with intronic snoRNAs. Numbers on the left indicate how many times each state was observed for eight intronic snoRNAs in 12 post-WGD species.

It has been observed that introns containing functional noncoding RNAs are usually well conserved across a broad evolutionary range (Chorev and Carmel 2013). Our analysis highlights examples where introns encoding snoRNAs have been lost. We therefore investigated the conservation of intronic snoRNAs in more depth, taking into account WGD. We found that after WGD, one genomic copy of the protein gene carrying intronic snoRNAs was retained in 64 out of 84 cases across all genomes, preserving both the intron and the snoRNA (fig. 7*A*). We found only 13 cases where an intron encoding a snoRNA was lost from the host gene. Only in the case of snR191 in the *NOG2* gene of *Naumovozyma dairenensis* did intron loss lead to snoRNA removal from the genome. *Kazachstania africana* has lost the same intron, but an additional copy of snR191 is present in a region non-paralogous to *NOG2*. In the five cases where the intron encoding the snoRNA was lost, the snoRNA was retained within the intron of the paralogous gene, and for another six cases a copy of snoRNA was present in the paralogous region but without the associated protein-coding gene (fig. 7*B*). This last mode of gene loss with retention of the intronic snoRNAs was previously described as “snoRNA deintronization” and has been shown to be common in the evolution of snoRNA genes in the Saccharomycetaceae (Mitrovich et al. 2010).

### Conservation of Ribosomal Protein Introns

Introns in RPGs have been implicated in maintaining the correct expression ratio of paralogous mRNAs in *S. cerevisiae* (Parenteau et al. 2011). It is well known that introns in ribosomal protein genes in *S. cerevisiae* are longer on average than introns of any other protein class (Neuveglise et al. 2011; Parenteau et al. 2011). We found 59 ancestral RPG introns in 56 cytoplasmic and 2 mitochondrial RPGs. The unusual nature of RPG introns is also reflected in the number of intron gains and losses: 105 losses for 59 ancestral introns across the 20 species, corresponding to 0.07 introns lost per locus in each species (compared with 0.21 for all introns). There are only two cases where the RPG appears to be lost from the genome—*MRPL44* in *T. blattae* and *RPL30* in *Lachancea waltii*. *MRPL44* is a mitochondrial ribosomal protein and *S. cerevisiae* knockout mutants are viable, although with decreased competitive fitness. The *RPL30* protein is essential in *S. cerevisiae* and additionally has a regulatory feedback loop residing on exon–intron boundary. How *L. waltii* compensates for the loss of this crucial 60S ribosome component remains to be discovered.

**Fig. 8.—:**
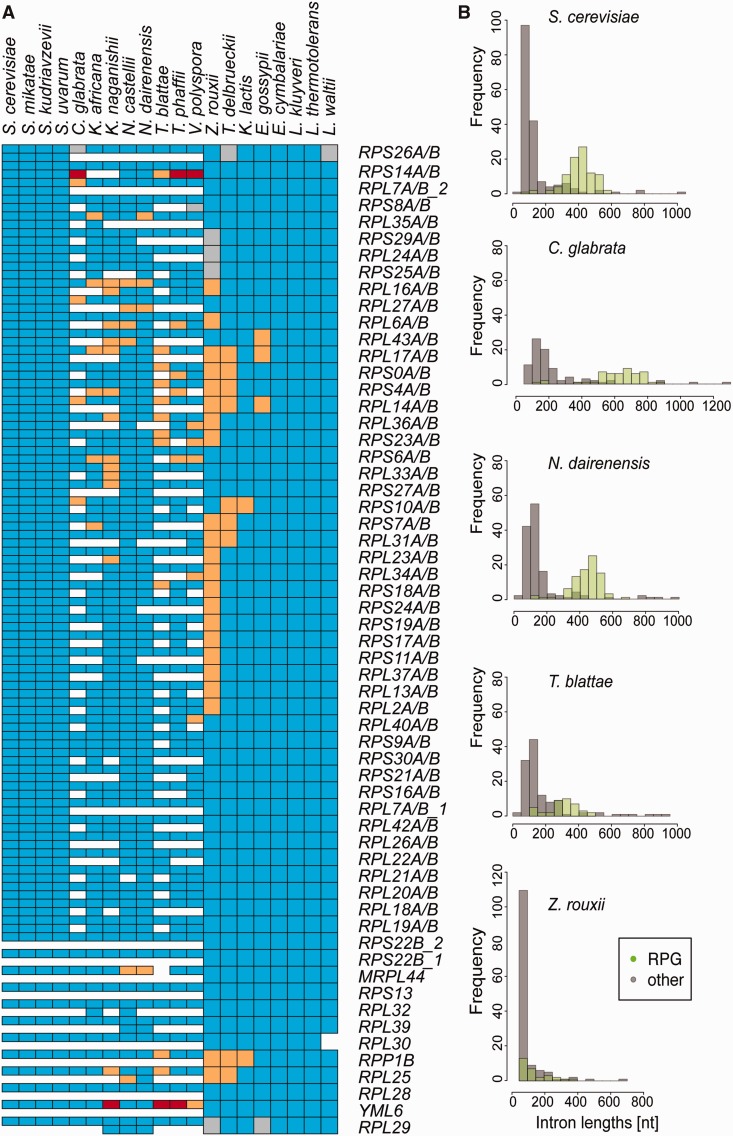
Introns in ribosomal protein genes. (*A*) Conservation of introns in RPGs. Each column represents one of the species listed above. Each row corresponds to an ancestral intron in ribosomal protein gene. For post-WGD species rows are divided in two to represent the presence of the duplicated copy. Legend: blue, intron present; orange, intron replaced with cDNA; red, intron removed with mutation; gray, intron not found. (*B*) Intron length frequency graphs for RPGs (green) and other genes (gray) in representative species. All species except *Z. rouxii* show some degree of bimodality in intron length distribution.

In the *S. cerevisiae* genome, 47 of the intron-containing RPGs have a paralogous copy. Interestingly, in *Saccharomyces* sensu stricto, paralogs of intron-containing ribosomal proteins always have the paralogous intron as well (fig. 8*A*). This result, together with the strong conservation of spliced transcript number and intron expression (fig. 2), suggests that the function of RPG introns is conserved across *Saccharomyces* sensu stricto*.* However, outside the *Saccharomyces* sensu stricto, there are multiple instances of the loss of one of the paralogous copies of a ribosomal protein gene. *Candida glabrata* and *T. blattae* show particularly striking patterns: In 51 and 48 out of 59 ancestral introns respectively, only one copy of the RPG is retained, but all retained copies preserve the intron. In contrast, *N. castellii* and *N. dairenensis* nearly always retain the duplicated ribosomal protein genes together with their introns. In other post-WGD species there is a varied degree of conservation of both ribosomal protein genes and introns. The only intron retained in both copies in 18 out of 20 genomes was in the paralogous *RPS9A*/*B* gene pair. *RPS9A*/*B* splicing has been reported to autoregulate the host genes not only in *S. cerevisiae* but also in *Drosophila melanogaster* (Plocik and Guthrie 2012), thus it is not surprising that the presence of both intron-containing *RPS9* copies is required in yeast. Among pre-WGD species, the most prone to lose introns from RPGs was *Z. rouxii*, with 26 introns missing out of an ancestral 59. It is also the only species where the number and length of RPG introns do not differ from other introns (fig. 8*B*). In summary, distinctive patterns for RPG intron conservation in different species lead us to conclude that the unusual characteristics of RPG introns are not shared by the whole Saccharomycetaceae, and are probably restricted to the *Saccharomyces* sensu stricto species.

## Discussion

### Intron Evolution Is Branch- and Species-Specific

This work represents a comprehensive evolutionary analysis of introns in the Saccharomycetaceae. It was previously proposed that hemiascomycetous fungi have undergone extensive intron loss (Stajich et al. 2007). The species in the Saccharomycetaceae continued to lose introns even after the divergence from CTG species and early branching species like *Y. lipolytica* (Neuveglise et al. 2011). We observed that specific species and branches have undergone additional intron loss compared with *S. cerevisiae*. *Candida glabrata* has experienced a particularly high number of intron losses and is an extremely intron poor species. This is consistent with the postulated reductive evolution of the *C. glabrata* genome, and the extensive loss of paralogous genes, both likely consequences of its pathogenic mode of life (Dujon et al. 2004). Intron loss has also been particularly high in some other clades, both that diverged before and after the WGD event: The *Zygosaccharomyces* clade (represented here by *Z. rouxii*) and the *Vanderwaltozyma* clade (represented by *V. polyspora)* (Souciet et al. 2009). In contrast to *C. glabrata* and Z. *rouxii*, which have undergone reductions in genome size, *V. polyspora* has a similar genome size and gene number to *S. cerevisiae* (Scannell et al. 2007). We therefore suggest that increased gene density is neither sufficient nor necessary for increased intron loss in yeast.

### Prevalent Intron Loss Provides Insight into Mechanisms of Deletion

Our results show that introns are usually lost with very high precision, with at most two codons inserted or deleted at the intron–exon boundaries. Both microhomology-mediated intron loss and reverse transcription of spliced mRNA and homologous DNA recombination (Mourier and Jeffares 2003) can lead to the replacement of intron-containing genes with their intronless versions. All yeast species exhibit a 5′-bias in intron positions (Bolotin-Fukuhara et al. 2006), a bias only explained by homologous recombination between cDNA and gene (Mourier and Jeffares 2003). We therefore assume that homologous recombination is responsible for the majority of intron losses that accompanied the divergence of yeasts from other fungi. Outside the *Saccharomyces* sensu stricto, individual species have lost between 15% and 46% of their introns, except for the *Lachancea* clade, where 7% of the introns tested appeared to have been lost on average. The most extreme case of intron retention is *L. kluyveri* for which we observe a total of nine introns missing, seven of which are probably intron gains in other species. Interestingly, *L. kluyveri* has undergone an unprecedented loss of the proteins mediating NHEJ and MMEJ pathways of DNA repair (Gordon et al. 2011). The closest relatives, *L. waltii* and *L. thermotolerans*, contain all genes involved in NHEJ/MMEJ, and we observed 13 events of perfect intron deletion. We therefore hypothesize that the inability of *L. kluyveri* to remove introns by replacement of the gene by the cDNA may be due to the loss of the NHEJ/MMEJ pathways. This observation is consistent with previously proposed models of precise intron loss mediated by NHEJ/MMEJ (Hu 2006; Farlow et al. 2011; van Schendel and Tijsterman 2013). Against the background of intron loss by homologous recombination in the yeast ancestor, the data therefore suggest that the microhomology-mediated end joining pathway may have contributed to intron loss within recent clades of hemiascomycetes.

Imperfect intron removal can alter the encoded protein and thus contribute to the landscape of protein evolution. We found 33 cases of insertion or deletion of more than three codons associated with intron loss—these might be due to an imperfect intron removal mechanism, such as degeneration of splice signals and subsequent deletions with preservation of the reading frame phase, or imprecise intron removal by NHEJ/MMEJ pathway. We observed intron sliding only in the *RPS22B* gene. Our results therefore indicate that imperfect intron removal events are rare, with only around 2.6% of intron losses resulting in addition or deletion of more than three codons.

### Intron Evolution Patterns after Whole-Genome Duplication

We analyzed in detail the evolutionary history of the eight intronic snoRNAs present in *S. cerevisiae*. Mitrovich et al. (2010) postulated that intronic snoRNAs have been “deintronized” in the *Saccharomycotina* clade through mutation of the boundary exons such that the snoRNAs have been conserved while eliminating the protein-encoding genes. Our evidence suggests that WGD can facilitate this process. In post-WGD species, protein-coding and intronic snoRNA genes have subfunctionalized, leading to retention of the intronless version of the gene at one duplicated locus, and retention of the snoRNA gene and loss of the protein-coding gene at the other. Besides deintronization of snoRNAs, single intron loss after duplication was also common, and other variations on modifying or decoupling protein and RNA genes were also observed (fig. 7).

Ribosomal protein genes appear to have a distinct mode of intron evolution. It has been previously noted that in *S. cerevisiae*, RPGs are enriched in introns and the introns are longer than for other classes of genes (Rodriguez-Medina and Rymond 1994; Spingola et al. 1999). The increased number and size of RPG introns have also been observed for *C. glabrata*, *K. lactis*, and *E. gossypii* (Neuveglise et al. 2011), and in all species besides *Z. rouxii* in this study, thus it is a feature that is pertinent to both pre- and post-WGD species. However, the degree of conservation of RPGs and introns varies among post-WGD species. We speculate that *Saccharomyces* sensu stricto retained most of the duplicated RPGs, and subsequently evolved a distinct mechanism regulating their expression. Accordingly, we observe a strict retention of RPG introns within *Saccharomyces* sensu stricto species. *Candida glabrata* and *T. blattae* may have coped with the duplication of RPGs by reverting to the preduplication state in which only one of the RPG paralogs is retained. All species usually maintain at least one intron-containing copy of each RPG, with the exception of *Z. rouxii*, which exhibited increased intron loss. *Zygosaccharomyces rouxii* is a halotolerant and osmotolerant species responsible for food spoilage. It was reported previously that *S. cerevisiae* introns contribute to the fitness in challenging stress conditions, so it would be expected that those introns would be kept in *Z. rouxii*, which is frequently exposed to stress concentrations of salt and sugar. We therefore hypothesise that *Z. rouxii* has developed other mechanisms to increase its tolerance to stress, and the greater loss of introns observed for *Z. rouxii* is due to relaxation or removal of the constraints on intron maintenance. We conclude that RPG introns have been preferentially kept in yeast genomes. It is likely that the introns perform different functions in different species. For example, in the *Saccharomyces* sensu stricto, the introns are probably regulating the level of RPG mRNAs, as reported by Parenteau et al. (2011), but in *Z. rouxii* their functional importance is decreased.

## Conclusions

Our multiple sequence alignments of orthologous introns in Saccharomycetaceae provide an unprecedented resource for the study of intron evolution in intron-poor genomes. Their analysis, together with RNAseq data, allows us to identify hundreds of previously unannotated introns in yeast species. We provide direct evidence for postulated mechanisms of yeast intron evolution. In particular, our data strongly support the prevalence of precise intron loss by homologous recombination of mature mRNA and genomic locus, but also NHEJ/MMEJ-mediated intron loss, in the recent history of Saccharomycetaceae. The complexity of organism-, gene- and intron-specific factors that affect intron fate are only just beginning to be understood.

## Supplementary Material

Supplementary table S1 and supplementary alignment information are available at *Genome Biology and Evolution* online (http://www.gbe.oxfordjournals.org/).

Supplementary Data
