# Supplementary material for: Intron Evolution in Saccharomycetaceae
Source: Genome Biol Evol. 2014 Sep 9;6(9):2543–56. doi: 10.1093/gbe/evu196 (PMC4202332; doi:10.1093/gbe/evu196)
Supplement: Supplementary Data [file supp_6_9_2543__index.html]

Intron evolution in Saccharomycetaceae — Intron Evolution in Saccharomycetaceae — Supplementary Data 

# Intron Evolution in Saccharomycetaceae

## Supplementary Data

files

**Files in this Data Supplement:**

- Supplementary Data - zip file
